# Towards a Secure Technology-Driven Architecture for Smart Health Insurance Systems: An Empirical Study

**DOI:** 10.3390/healthcare11162257

**Published:** 2023-08-10

**Authors:** Fatima Al-Quayed, Mamoona Humayun, Sidra Tahir

**Affiliations:** 1 Department of Computer Science, College of Computer and Information Sciences, Jouf University, Sakakah 72341, Saudi Arabia; 2 Department of Information Systems, College of Computer and Information Sciences, Jouf University, Sakakah 72311, Saudi Arabia; 3 University Institute of Information Technology, PMAS Arid Agriculture University, Rawalpindi 43600, Pakistan; stahir@uaar.edu.pk

**Keywords:** smart health insurance system, technology-driven, 5G, cloud, blockchain, machine learning

## Abstract

Health insurance has become a crucial component of people’s lives as the occurrence of health problems rises. Unaffordable healthcare problems for individuals with little income might be a problem. In the case of a medical emergency, health insurance assists individuals in affording the costs of healthcare services and protects them financially against the possibility of debt. Security, privacy, and fraud risks may impact the numerous benefits of health insurance. In recent years, health insurance fraud has been a contentious topic due to the substantial losses it causes for individuals, commercial enterprises, and governments. Therefore, there is a need to develop mechanisms for identifying health insurance fraud incidents. Furthermore, a large quantity of highly sensitive electronic health insurance data are generated on a daily basis, which attracts fraudulent users. Motivated by these facts, we propose a smart healthcare insurance framework for fraud detection and prevention (SHINFDP) that leverages the capabilities of cutting-edge technologies including blockchain, 5G, cloud, and machine learning (ML) to enhance the health insurance process. The proposed framework is evaluated using mathematical modeling and an industrial focus group. In addition, a case study was demonstrated to illustrate the SHINFDP’s applicability in enhancing the security and effectiveness of health insurance. The findings indicate that the SHINFDP aids in the detection of healthcare fraud at early stages. Furthermore, the results of the focus group show that SHINFDP is adaptable and simple to comprehend. The case study further strengthens the findings and also describes the implications of the proposed solution in a real setting.

## 1. Introduction

In recent years, the number of individuals with health insurance (HIN) has expanded considerably around the globe. In industrialized nations such as the US, more than ninety percent of inhabitants have health insurance. HIN promotes access to care and is connected with decreased mortality rates, improved health outcomes, and enhanced productivity [1,2]. Those who do not use HIN endanger their physical, emotional, and financial wellness. Meaningful healthcare coverage is essential to live a productive, secure, and healthy life [3,4]. HIN is accessible via a range of private and public sources. It is a contract between the health insurance provider (HIP) and the health insurance subscriber (HINS) in which HIP bears the healthcare expenses of HINS. HIN covers ordinary healthcare concerns and losses due to accidents, healthcare expenditures, incapacity, unintentional injury, and damage. However, in order to obtain this benefit, HINS must constantly pay the fee for this compensation [1,4,5]. A high uninsured rate strains the whole healthcare system. People without HIN delay necessary treatment and depend more heavily on hospital emergency rooms, diverting limited resources to address diseases that might often have been averted or treated in a lower-cost environment. The absence of HIN has severe cost consequences for people, communities, and the health care system [6,7,8].

**Figure 1:**
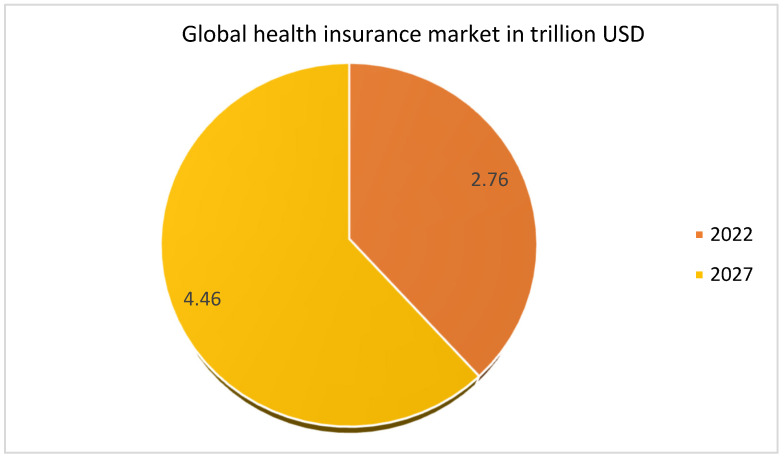
Global HIN market prediction in USD trillions.

Previously, the health insurance claim (HIC) procedure was semi-automated, with several drawbacks, such as wasted time and money and the HINSs’ need to frequently visit the HIN office to initiate the premium request and to know about HIC status. Furthermore, auditing of HIC requires more human resources and is more prone to mistakes as handling paper-based HIC data is arduous. In such a system, HIC documents are modifiable and freely available. Consequently, the HIP, healthcare service provider (HSP), and HINS are vulnerable to fraud owing to a lack of privacy and transparency. Nowadays, information is collected in a digital format, revolutionizing HIC globally. The advantages of digitization include convenience to the parties engaged in HIC, no need for manual visits, fast communication between HINS and HIP, audit and checking, lack of fraudulent activities, low cost, easy claim verification, and many more [9,10,11]. The digitization and benefits associated with HIN have motivated individuals worldwide to purchase appropriate HIN policies. The global HIN market is increasing rapidly and is predicted to increase further, as can be seen from the statistics in Figure 1. The statistics used in Figure 1 are taken from the “Research and Market” report [12].

Despite several advantages, the digitized HIN market has to face numerous challenges, and one of the critical challenges faced globally is HIN fraud. Vast sums of money are lost worldwide due to HIN fraud [13], raising HIP firms’ administrative costs and the premiums HINS must pay [14]. A total of around GBP 303.8 million was lost to HIN fraud, according to a 2015 study on the financial impact of HIN fraud [13]. This sum may be divided into GBP 237 million for fraudulent pharmaceutical charges, GBP 43.9 million for fraudulent dental expenses, and GBP 22.9 million for fraudulent optical charges. Similarly, Thaifur et al. [15] estimate that healthcare fraud costs Korea and Europe, respectively, EUR 798.2 billion and EUR 56 billion annually. The monthly medical assistance payments for each member of the national HIN in South Africa increased by ZAR 192 (USD 14) to ZAR 410 (USD 30) due to fraud. These minor healthcare overheads are projected to be about USD 882 million.

Since the last decade, cutting-edge technologies, especially AI and blockchain, have been widely used in the healthcare sector for fraud detection and prevention [3,16,17]. By analyzing previous data, AI can fuel prediction models that evaluate the likelihood of an occurrence happening. Not only that, but they may also estimate the worth of the HIC by analyzing past claims made by the policyholder. A claim value above the predicted value can be forwarded for further investigations [18,19,20,21]. In the same way, end-to-end traceability and a smart contract in the BLC help to detect and prevent HIN fraud [5,22,23,24,25].

On the other hand, 5G facilitates the fast transmission of insurance data between different entities. At the same time, the cloud overcomes the limited capacity of the blockchain digital ledger (DTL) and provides a secure storage mechanism for healthcare data. Considering the importance of HIN globally, this research leverages the potential of the above-mentioned cutting-edge technologies to propose a smart solution for HIN fraud detection and prevention. The problem statement and the explicit contribution of the study are mentioned in the following subsections

### 1.1. Problem Statement

Policyholders frequently give false or deceptive information to health insurance companies in an effort to obtain reimbursement for unapproved benefits, which results in a significant loss to the HINP. A transparent end-to-end processing method for HIN claims is required in order to help identify and stop HIN scams.

### 1.2. Paper Contribution

The explicit contributions of this study are listed below:

1. We explore the existing literature to identify possible HIN fraud and the entities involved in it;
2. We provide a review of the various current usages of cutting-edge technologies for healthcare applications, especially in the HIN process;
3. We propose a layered framework based on cutting-edge technologies to detect and mitigate HIN frauds;
4. The proposed framework is evaluated using mathematical modeling, and an industrial focus group;
5. The practical implications of the proposed framework are elaborated with the help of a case study.

Before proceeding to the background and overview of existing studies, Acronyms part in Abbreviations lists the study’s acronyms and their complete forms.

**Figure 2:**
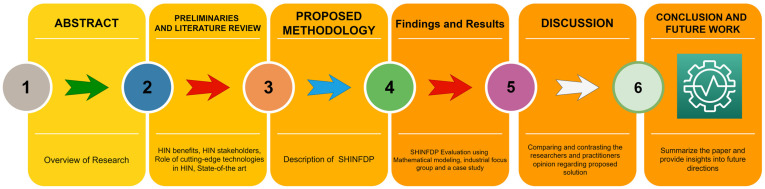
Study roadmap.

Figure 2 depicts the structure of the remaining paper.

## 2. Preliminaries and Related Work

This section will provide an overview of HIN, the challenges faced by HIN, and the role of cutting-edge technologies in HIN.

### 2.1. Why There Is a Need for HIN

Having HIN improves access to care, which leads to fewer deaths and improved health outcomes. HIN may represent the difference between disease and health, or life and death. One of the primary causes of mortality, particularly in emerging and underdeveloped nations, is a lack of HIN. Healthy individuals and young adults may believe they do not need HIN since they are seldom ill. Accidents and diseases, on the other hand, may happen to anybody at any moment. Without HIN, we are responsible for funding our own medical expenditures, placing us in severe financial jeopardy [26,27,28]. Therefore, HIN is vital to living a healthy and peaceful life. Below are some key benefits of HIN.

**Financial benefits**

The following financial benefits are associated with HIN:

- HIN aids in the payment of medical costs and prescription medication costs.
- It assists you in avoiding significant medical debt.
- It limits how much you may spend on healthcare each year.

**Health benefits**

People usually do not visit a healthcare center regularly without HIN. Individuals with HIN have the following benefits:

- HIN helps you live a longer, healthier life.
- It connects you to a consistent source of care.
- It aids in the diagnosis and treatment of ailments and disorders.

**Peace of mind**

An essential thing in an individual’s life is peace of mind; however, the chances of illness are always there. Most people are not only afraid of disease but also more fearful of the cost they have to bear. HIN provides peace of mind in the following ways:

- Reducing financial worries regarding medical expenses.
- Reducing health worries regarding a lack of access to care.
- Removing the stigma associated with being uninsured.

### 2.2. Healthcare Insurance Claim (HIC) Processing

**Figure 3:**
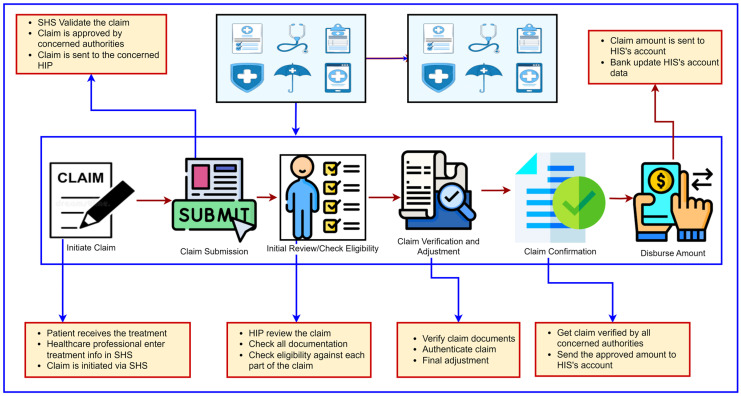
HIC processing steps.

The HIC processing cycle needs to be understood to better understand HIN fraud. Various stakeholders are involved in processing HIC, such as doctors, paramedical staff, hospital administration, HIN subscribers, and HIP [29,30,31]. The hospital administration initiates the claim when a patient receives treatment from the hospital; all of the HSPs involved in the treatment enter the treatment information into the smart healthcare system (SHS). The HSP personnel who is responsible for preparing HIC makes a claim and has it verified by the concerned HSP authorities. Once the HSP verifies the claim, it is sent to the HIP for further processing. When the HIP receives a claim, it verifies it and checks the patient’s eligibility and claim validity. Once the HIC is verified and validated, it is sent for approval in the concerned section. Once the HIC is approved, the amount of HIC is transferred to the HSP or HINS’s account. The complete steps of HIC process are provided in Figure 3.

### 2.3. Healthcare Insurance Fraud

**Figure 4:**
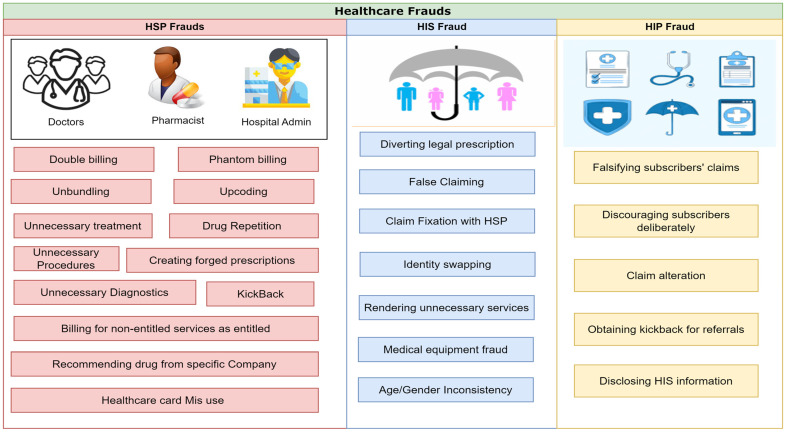
Taxonomy of HIN frauds.

HIN fraud impacts people and companies equally, costing tens of billions of dollars annually. It may result in higher HIN rates, more invasive medical treatments, and more taxes. HSP, HINS, HIP, and other parties that purposefully mislead the healthcare system to obtain illegal benefits or payments may engage in HIN fraud [16,20,22,23,24]. Frequently reported forms of HIN fraud are depicted in the taxonomy of Figure 4. Below, we discuss these forms of fraud in detail.

#### 2.3.1. Healthcare Service Provider (HSP) Fraud

HSPs are the professionals involved in providing and managing healthcare services. These professionals include doctors, hospital administration, ambulances, and laboratories. HSP involvement is a prime factor that facilitates most HIN frauds, even those that HINSs commit. Some common HIN frauds committed by HSPs include: (1) Double-billing—In a double-billing scam, an HSP submits numerous claims for the same set of services. It is common for HSPs to double-bill the same entity (such as the government) for the same set of services. They may obfuscate their activities by changing the service’s date or description, or the patient’s or provider’s identity. (2) Phantom billing—Phantom billing is invoicing the HIN or the government for services that were never provided. (3) Unbundling—This is a kind of fraudulent activity in which doctors charge for each stage of a multi-part process instead of lumping them together under one code. By separately billing each phase, the HSP may earn more money while still providing the same level of care. Unbundling, or “fragmentation,” is breaking up a single medical operation into many codes to increase individual payments. The medical coding industry often engages in unbundling. Instead of utilizing the normal billing code, a surgeon who participates in unbundling could input different codes for incision and suturing during a regular surgical operation. As a result, a doctor will receive more money. (4) Upcoding—Using a more expensive billing code for the same medical service is called “upcoding” or up-charging. This means that the HSP is charging patients for a greater quality of service than they are providing. The HSP falsely increases their remuneration by upcoding the severity or complexity of the patient’s condition or treatment. For financial gain, an HSP may sometimes misdiagnose their patients’ conditions, such as diagnosing with chronic bronchitis when they have acute bronchitis or with a minor skin lesion when they actually had a bigger, more complex excision. (5) Unnecessary treatment/drug repetitions—In this fraud, the doctor continues the treatment/drugs for a long time without needing them. (6) Unnecessary procedures/diagnostics—Sometimes, doctors recommend costly procedures/diagnostics that are not required (e.g., recommending a biopsy treatment for the patient with a temporary disorder). (7) Creating forged prescriptions—HSPs counterfeit or fraudulently modify or create a prescription or other document in this scam. Two examples are adding a medicine or changing the date, amount, or dispensing instructions. (8) Kickback—This is a term used to describe a misuse of money that benefits a person of authority to use his position or influence to benefit another individual, organization, or enterprise. It is a prevalent deception in the HIN area, where HSPs such as pharmaceutical corporations incentivize other HSPs to suggest certain drugs. (9) Billing for non-entitled services as entitled—This fraud usually happens when an HINS is not entitled to a particular service by the HIP and the doctor provides him this service, putting this treatment in another category entitled to him.

#### 2.3.2. Healthcare Insurance Subscriber (HINS) Fraud

The HINS is the one who purchases HIN policy from the private- or public-sector organization. Most HIN fraud comes from HINSs seeking to obtain financial benefits. HINSs perform HIN fraud in different ways: (1) The HINS may change prescriptions by himself or with the help of an HSP. (2) Sometimes, HINSs make false claims and try to receive money for a treatment they never received. (3) Another method of HIN fraud is claim fixation, in which both the HINS and HSP are involved, modifying the claim amount to obtain mutual financial benefits. (4) One of the most commonly occurring types of fraud that significantly affect HIPs is identity swapping, in which an HINS’s insurance card/policy is used by someone else to access healthcare facilities. (5) HINSs sometimes receive unnecessary services due to the availability of HIN (e.g., the HINS may visit the HSP and demand unnecessary diagnostics). (6) Another fraud committed by HINSs is medical equipment fraud, in which the HINS tries to obtain something more than what is required (e.g., the HINS needs a manual wheelchair, and he applies for a fully-automated wheelchair). (7) Age/gender inconsistency is another form of fraud, in which an HINS tries to hide his age/gender to obtain additional benefits for him or someone else. These are the possible methods of fraud initiated by the HINS. (8) Last but not least is the misuse of a healthcare card, in which a person uses his healthcare card for his family members or friends with the consent of the HSP and obtains the treatment cost from the HIP.

#### 2.3.3. Healthcare Insurance Provider (HIP) Fraud

HIPs conduct HIN fraud by misleading, hiding, or distorting information, although not to the level that HSPs and HINSs are engaged in healthcare fraud. The reason for not being involved in HIN fraud is to maintain the reputation of the HIP’s company and customer attraction. Still, some HIP fraud occurs when an impostor HIN agent attempts to sell you a false HIN plan or when a fraudster contacts an HINS to verify personal information for his plan and then steals the HINS’s identity. HIN firms may sometimes perpetrate fraud by fabricating medical equipment claims, particularly for older people or persons with disabilities, who often need a large amount of durable medical equipment. Sometimes, an HIP deliberately delays the claim processing and expects some incentive for the quick processing of HIC.

The above explanation demonstrates that HIN fraud may be conducted by HSPs, HIPs, HINSs, and others who purposefully defraud the HIN system to earn illegal benefits or payments. Hence, there is a need to investigate HIN fraud and provide a solution by using cutting-edge technologies. In the subsequent sections, we explore the role of cutting-edge technologies in healthcare fraud detection.

### 2.4. Role of AI in Healthcare

AI approaches have shown to be an effective tool for detecting HIN fraud. The HIN fraud detection system may be automated using AI. According to recent research, AI has primarily been utilized to identify HIN fraud using various techniques and models [32,33]. Anomalies and fraud are detected using behavioral profiling approaches based on ML algorithms. To this end, each individual’s behavior is modeled to detect deviations from norms [34,35]. HIN fraud detection ML approaches are classified into supervised learning (SL), semi-supervised learning (SSL), and unsupervised learning (USL).

A dataset of previously known fraudulent and valid records is used in the SL approach in HIN fraud detection. These data are used to capture fraud patterns and construct the model. The primary advantage of the SL approach is that the classification results produced by this technique are simple to understand. Many classifications and SL regression analysis methods are applied in HIN fraud detection. SL techniques used to detect HIN fraud include neural networks, decision trees, statistical analysis, Bayesian approaches, SVM, graph analysis, and rule-based systems [36,37]. Apart from its advantages, SL has several disadvantages. Data collection and data production are complex in SL approaches. When the HIN dataset is relatively large, data labeling becomes more complicated. It is difficult to distinguish between labels in fraud data when they are imprecise and ambiguous. Because of these limitations, SL implementation might be difficult in some instances. These SL limits may be overcome with the aid of USL.

USL algorithms detect fraudulent HIN activities in unlabeled HIN datasets. The absence of tagged data is an advantage of HIN fraud detection via USL [38,39]. When labeled data is lacking, USL is utilized to identify HIN fraud. HIN fraud detection employs various USL approaches, including association rules mining, k-means clustering, data mining, and k-nearest neighbor. SSL combines the advantages of both SL and USL. Compared to unlabeled data, hybrid SSL is employed when a limited amount of labeled data is available. In SSL, a predictive model is developed utilizing both labeled and unlabeled data [40]. In HIC fraud detection, combined clustering and classification as an SSL approach is often utilized.

### 2.5. Role of Blockchain in Healthcare

A BLC is a network of interconnected blocks that serves as a digital ledger (DTL) of transactions [41]. Each block contains a timestamp, block number, block header, version, nonce, transaction data, and a cryptographic hash of the preceding block. A Merkle tree is used to preserve transaction data [42]. In a BLC, if any of the transactions in a block are changed or amended, the hash value of that specific block will be drastically affected. It also disrupts the BLC, making it easier to spot modified transactions. As a result, once a transaction has been put to the BLC, it cannot be changed or amended. Data on the BLC is thus unchangeable. HIN applications benefit from BLC as a decentralized architecture since it enables distributed HIN application activities independent of a centralized server. Transparency among all parties engaged in the HIN process is created via the replication of DTL data across all nodes [36]. Due to the fact that the data in the DTL is duplicated across all nodes in the network, an attack that takes place on any one node in the BLC network has no effect on the state of the DTL. The preservation of unalterable HIN data is one of the prerequisites for an HIN fraud detection system [43,44]. The BLC’s immutability characteristic satisfies the aforementioned need. It is essential to maintain the validity and integrity of the HIC records. Cryptographic methods are used to encrypt the HINS’s data on the BLC, ensuring that only an HINS with legitimate authority may access and decode the data. It significantly improves the HIN’s data security and privacy. Since the identities of HINSs on a BLC are preserved using cryptographic keys, HINS data may be shared among all parties engaged in the detection of HIN fraud without disclosing the names of the HINSs [45]. BLC technology enables smart contracts, which are used to control activity after an agreement and are written as executable code, developed in Solidity language and implemented on Virtual Machines [46]. The whole HIN business process is governed by the transaction logic included in it. Once the transaction saves the operation, smart contracts are put into effect. In order to govern how their HIN data are shared or utilized in the network, HINSs may specify rules for the HIN process using a smart contract.

The working and roles of cutting-edge technologies in HIC processing and fraud detection are discussed in the above sub-sections; now, we explore the existing studies on using cutting-edge technologies for HIC fraud detection and prevention in the following subsection.

### 2.6. Role of 5G and Cloud in Healthcare

Fifth generation (5G) is a term referring to wireless network technology, and its use in the healthcare industry has created new opportunities for medical innovation and increased access to treatment. It is an air interface that is unified and powerful and was developed with an improved capacity to enable the user experiences and services of the future generation. The technology of 5G networks is one of the important technologies for the digital transformation of society [47,48,49]. It is also a requirement for the interconnectedness of everything involved in smart healthcare. It is possible to decrease discrepancies in the allocation of medical resources and hasten medical developments via the promotion and implementation of 5G smart healthcare [50]. The use of cloud computing in the healthcare industry makes the exchange of medical records simpler and more secure, automates back-end processes, and even makes it easier to develop and maintain telehealth applications. The healthcare business can reduce expenses while improving operational efficiencies by using cloud computing [51,52]. The performance of the healthcare business will be improved by 5G when combined with the cloud since cloud-sharing of large files will become quicker and more efficient with 5G.

### 2.7. Related Work

This section will provide an overview of recent studies that have used AI or BLC or both to identify and mitigate HIN fraud to provide a picture of the state of the art.

A rigorous assessment of AI- and BLC-enabled secure HIN fraud detection is presented by Kapadiya et al. [17]. This study offers a taxonomy of numerous HIN security challenges and a BLC- and AI-based safe and intelligent system for detecting HIN frauds. A case study involving HIN is also included to provide an overview of the proposed approach. Finally, it discusses outstanding difficulties, research hurdles in BLC implementation, and an AI-powered HIN fraud detection system.

According to Saldamli et al. [5], HIN firms are utilizing an antiquated approach to keep track of their client’s health care data and insurance-authorized information. As a result, several scams may occur in the HIN domain. The consumer attempts to claim the HIN from two distinct parties by defrauding the HIN businesses. As a result, there is a need to develop a secure system to monitor all of a user’s HICs to identify fraud. This article proposes a solution prototype that eliminates intermediaries and makes use of BLC technology to store and track data. Once data is written on a BLC, it cannot be changed or erased; this aspect of BLC ensures data integrity while identifying fraud. If any modifications are made to the BLC node, all further changes must be performed on the other nodes. BLC also maintains a record of log actions, which administrators may examine at any time. Despite its drawbacks, according to research results, BLC technology is one of the most popular strategies utilized today in the healthcare field. As a result, BLC enables us to solve many of the issues faced by the healthcare business, such as data integrity, data security, and fraud detection.

According to Ismail and Zeadally, HIN fraud detection is a big challenge for healthcare [24]. HIN fraud costs billions of dollars each year. Given the significance of the issue, this paper proposes Block-HI, a BLC-based framework for detecting HIN fraud based on a proposed taxonomy of 12 different fraud scenarios. When the number of HIN branches and HICs transactions increased, the performance of Block-HI was tested in terms of execution time and total quantity of data sent for ledger updating. The results show that when the number of HIN branches rises, the execution time of Block-HI decreases by just 0.69% on average, although the number of claims increases by 33.51%. This is because the consensus procedure was executed. Despite the fact that performance degrades as the number of branches grows, the suggested framework, which automates fraud detection, can uncover distinct fraud situations compared to a human procedure.

Amponsah et al. suggest a method for detecting and preventing fraud in HIC processing utilizing ML methods and BLC technology [53]. The original HIC dataset is classified using a decision tree in this work. The retrieved information is encoded in an Ethereum BLC smart contract to identify and prevent healthcare fraud. According to the trial data, the top-performing tool has 97.96% classification accuracy and 98.09% sensitivity. The study’s results demonstrate that the suggested solution improves the capacity of BLC smart contracts to identify fraud with an accuracy of 97.96%.

According to Gera et al., several concerns are linked to the HIN sector, such as fake HICs and claims unlawfully influenced by competent authorities [54]. This article addresses these concerns by building a BLC-based HIN application. The consensus guarantees that the HIN company’s claim procedure is carried out securely. In particular, every transaction in the proposed approach is cryptographically signed and kept on the BLC as a collection of blocks. This method protects HIC transactions and prevents any fraudulent efforts. The IBM BLC technology and its underlying components are used to create a prototype application. The experimental findings demonstrated that the suggested implementation reduces HIC fraud.

According to Goyal et al., having health insurance is vital for everyone, especially with rising medical expenditures, since medical crises may have a significant financial and emotional effect [55]. However, the existing insurance system is too costly, and the claim settlement procedure is overly protracted and cumbersome. Consequently, policyholders cannot file an HIC with their insurance carrier. The authors of this study provide a rapid and cost-effective framework for an HIN business using BLC technology and ML. By constructing a smart contract, BLC may eliminate intermediary organizations, making the whole process easier, safer, and more efficient. The smart contract pays the HIC based on the HINS’s documentation. A ridge regression model is used to best compute premiums based on the total amount claimed during the current policy duration and various other criteria. A random forest classifier is used to anticipate risk, which aids in the calculation of risk-rated premium rebates. The suggested model’s findings demonstrate that it is dependable, cost effective, and rapid. The whole HIC processing method with fewer intermediaries and smart contracts makes the procedure faster, and risk-rated premium rebates assist in incentivizing those with lower claims to keep their insurance. It also prevented the additional cash created in the present HIN system by dispersing it in risk-based rebates. Results also reveal that the random forest classifier delivered higher accurate results out of the numerous multi-class classification ML approaches.

Amponsah et al. suggest a BLC-based method to improve the financial sustainability of HIN systems [56]. Poor data management, lengthy lines of communication, lack of transparency among stakeholders in HIC processing, fraud, and corruption are the issues confronting the HIN and jeopardizing its viability. This paper suggests a cloud-based BLC approach to overcome these issues. According to the findings of this work, there is a clear demonstration of communication line reduction, efficient management and storage of data using the cloud, efficient HIC processing lifecycle, restricted data access, hypothetical reduction in HIC processing, security, and high transparency in the system. After evaluating the system’s read and write operations, it can be concluded that it accomplishes its goals since all activities were completed in the world state and BLC systems, respectively. The study also emphasizes the significance of information, service quality, and user happiness in effectively implementing the cloud BLC-based solution.

The above discussion shows that cutting-edge technologies play vital roles in e-health and HIC processing. However, existing studies mainly focus on improving HIN acceptability and the security of HIN systems. Furthermore, existing studies mainly focus on a particular technology instead of leveraging the potentials of combination of technologies. We did not find any study that explicitly addresses fraud detection and mitigation in the end-to-end HIC processing cycle. To address this gap, we propose an end-to-end security model for HIN fraud detection using cutting-edge technologies in the next section.

## 3. Proposed Methodology SHINFDP

**Figure 5:**
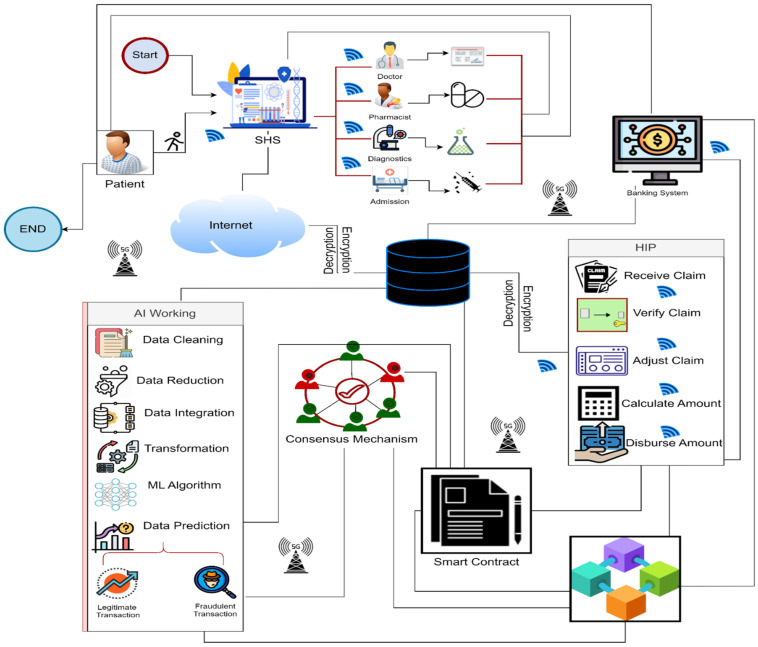
Proposed methodology.

To address fraud detection and mitigation for HICs, we proposed an end-to-end layered security framework named SHINFDP, as shown in Figure 5. The proposed SHINFDP consists of six layers: user layer, communication layer, storage layer, prediction layer, security layer, and HIC processing layer. According to SHINFDP, a patient approaches the healthcare center (HC) online or physically. The patient’s query is considered by the relevant staff in the HC, and the particulars of the patient are stored in SHS. The patient is referred to the relevant HSP based on the stored particulars. The HSP provides the treatment to the patient and adds the details of treatment in the SHS, from which another HSP accesses the patient’s details for preparing the HIC. Once the HIC is prepared, it is stored in the cloud, from which the prediction layer accesses it. The prediction layer ensures the claim’s validity and stores it in the BLC. The HIP accesses the HIC through the prediction layer and performs the necessary actions. The transactions’ details are stored in the smart contract of the BLC; each layer of SHINFDP communicates with the BLC using the smart contract. Below, we describe the functionality of each layer.

### 3.1. User Layer

Three main users are involved in the proposed SHINFDP, including the HIP, HSP, and HINS. The HINS is the insured patient who initiated the system by approaching the HC for treatment. The insured patient can visit the HC in person or may contact the HSP using a smart healthcare system (SHS). The HSP concerned with handling patients assesses the HINS, checks his insurance policy, and sends him to the relevant HSP for receiving treatment. The HSP includes various healthcare professionals (e.g., doctors, paramedic staff, diagnostics/lab staff, pharmacist, etc.). The HSP performs the treatment according to the patient’s situation and enters the treatment details in the SHS, from which the HSP concerned with HIC processing fetches the data and prepares the HIC. The prepared HIC is approved by the relevant authorities and is stored in the healthcare cloud using a proper encryption mechanism. The prediction layer responsible for detecting legitimate HICs and fraudulent HICs provides classification of the HIC and stores the legitimate HIC in the BLC after the execution of the BLC consensus mechanism. A smart contract is created for all the legitimate HICs. The HIP accesses the legitimate HIC and processes it according to the patient’s HIN policy. The claim record is also stored in the BLC’s digital ledger (DTL), from which each party can access it. The user layer uses the services of various other layers; this layer uses 5G services for fast data transmission between multiple entities. Furthermore, this layer is connected to a storage layer, where the HIC is stored for further processing. This layer is also associated with the security layer, as all user records are stored in the BLC. The details of the tasks performed by all three categories of users are outlined in Algorithm 1.
**Algorithm 1:** Working of user layerLet ρ=HIS appraoches HC physically; HC = Healthcare Cloud E = Encryption; CA = Claim amount; LHIC = Legitimare health insurance claim **Begin**  1.$HIS\to HC⇑\left(5G,\rho \right)$  2.$HSP\to EnterRecord\left(HIS\right)\to SHS$  3.$HSP\to Refer\left(HSP\right)\to T$  4.$HIS\to Recieve\left(T\right)\to HSP$  5.$HSP\to EnterInfo\left(T\right)\to SHS$  6.HSP→Prepare(HIC)  7.$HSP\to Validate\left(HIC\right)\to HSP$  8.$HSP\to Forward\left(HIC\right)⇑\left(5G,E\right)\to HC$  9.*Add HIC* →*Blockchain*  10.$HIP\to Recieve\left(LHIC\right)$  11.$HIP\to Process\left(LHIC\right)$  12.$HIP\to Dispatch\left(CA\right)\to \left(HIS,HC\right)$**End**

### 3.2. Communication Layer

This layer leverages the potential of modern 5G technologies to provide fast data transmission between multiple layers. As proposed, SHINFDP provides end-to-end security for HIC processing; therefore, 5G services are vital as they will provide massive capacity, low latency, and high speeds. The HINS will use 5G to communicate with the HSP and HIP and to track the HIC. The HSP will use 5G to transmit and store patients’ data and the corresponding HIN policy. Furthermore, 5G will be involved in data transmission between the SHS and the cloud. The prediction layer will also use 5G services to fetch and analyze data. The security layer will use 5G’s potential to execute consensus mechanisms, generate smart contracts, and manage the BLC network. The HIC processing layer will use 5G potentials to fetch legitimate HIC for processing and dispatching the HIC amount to the concerned authority. Algorithm 2 elaborate the working of communication layer.
**Algorithm 2:** Working of communication layer1:Input: Data D, Source address S, destination address T2:Output: Find best routing path with low latency LL and High speed HS3:Procedure4: Buffer = 0a.If (buffer <= buffer.Length) thenb.Receive (D,S,T)c.Find best routing path(↓LL,↑HS)d.transmission(D,S,T)e.elsef.empty(buffer)g.Go to Step ch.End ifEnd Procedure

### 3.3. Storage Layer

This layer of SHINFDP leverages the potential of cloud computing to store HIC data on the cloud securely. As the proposed SHINFDP is smart, the data collection mode will be online only. All the data generated by the HSP will be stored on the SHS. The HSP responsible for HIC generation will fetch data from SHS, prepare the HIC, and store it on the cloud. The data generated by this layer include payment-related sensitive information of the HINS and HIP so that it will be shared and controlled through smart contract conditions for security considerations. The prediction layer will fetch data from this layer for classifying legitimate and fraudulent HICs. The HIP will fetch the data of legitimate HICs from this layer for further processing. Working of storage layer is elaborated in Algorithm 3.
**Algorithm 3:** Working of storage layer1: Input: HIC Data

2: Output: Secure storage of data in the cloud *and Blockchain*

3: Procedure

 HSP→FetchData←SHS

 Generate (HIC)←SHS

 Validate(HIC)

 SmartContract(HIC)

 HIC→BlockChain

End Procedure


### 3.4. Prediction Layer

This layer of the proposed SHINFDP will leverage AI’s potential to classify HICs into legitimate and fraudulent HICs. This layer will fetch HIC data from the cloud and will clean the data for redundancy, missing values, and incorrect information. The process of preparing data for analysis by deleting or changing data that is erroneous, incomplete, irrelevant, redundant, or badly formatted is known as data cleaning. In order to clean the data, suitable data cleaning technique of ML will be used. Data cleaning is one of the important steps in ML; this process will detect missing or null values, outliers, and erroneous data. The claim amount range for each treatment will be determined and saved in the healthcare cloud repository. To identify outliers, the data’s lower and upper bounds will be computed. Outliers are values that are both below and above the lower and upper bounds. This will be accomplished by employing the appropriate ML algorithms to find outliers in the data. The cleaning process will reduce the data size; after that, data from different sources will be integrated and transformed into a uniform format for preprocessing. Once the data is transformed, ML algorithms will be applied to this data to classify it into legitimate and fraudulent data. The techniques used at this layer can be SL, USL, or SSL, depending on the nature of the data. AI data analytics is the key to minimizing the frequency of HIN fraud detection and the expense of false claims filed against HIN policies. The working of prediction layer is elaborated in Algorithm 4.
**Algorithm 4:** Working of prediction layer1: Input: HIC Data

2: Output: Classification of claim

3: Procedure

 FetchData (HIC)←Cloud

 CleanData(HIC)

 PreProcessData(HIC)

 TransformData(HIC)

 ApplyMLalgo

 Classsify(HIC)→(Legitimate, malicious)

End Procedure


### 3.5. Security Layer

The security layer is one of the critical layers of SHINFDP, which leverages the potential of BLC to prevent improper manipulation of the HIC. BLC is a series of blocks, each of which is nothing more than an encrypted real-world transaction. It is a transactional DTL that is open-source and community based. It is a peer-to-peer mechanism that guarantees openness and accountability. Each transaction is stored as a block in the DTL and may be programmatically validated by numerous users connected with the application. Every node has access to a copy of the BLC. It is built such that no one entity or node may conduct fraud. Every node utilizes the same BLC, and no conflicting versions are possible. There is a continual verification process with mining, and the consensus intends to provide evidence for every transaction. This technology is unusual in that it can keep data immutable and does not rely on any central authority. As a result, it maintains transaction integrity and may effectively prevent malpractice or hacking. It can also replace conventional organizations while providing a greater degree of security. The HIC procedure involves many parties, including HIP, HINS, and HSP. Each participant will have a peer node in the BLC’s peer-to-peer network. The HIP utilizes the HSP peer, which is responsible for HIC processing. The prediction layer requires a peer to complete the verification procedure. An HSP peer may help with claim development and verification. All of these peers will be allowed to participate in HIC processing, and the transactions will be stored in a DTL. Each transaction is digitally signed and recorded on the BLC. In a distributed context, an irreversible collection of transactions is therefore preserved. Furthermore, smart contracts are generated for each transaction and are stored on the BLC. A smart contract allows each layer of SHINFDP to interface with the BLC layer. Smart contracts are programs for the authentication and payment of HIN coverage and are automatically executed when all the conditions are met. Consensus algorithms are used in smart contracts to authenticate every transaction and input. A smart contract is connected with the IPFS to reduce total data storage and quick data retrieval costs. The benefits of using a smart contract for HIN fraud detection include transparency, task automation, time saving, protection of HIN policy documents, and risk assessment. The detailed working of the security layer is outlined in Algorithm 5.
**Algorithm 5:** Working of security layerLet ST = Smart Contract; HCl = Healthcare Cloud; E = Encryption; CA = Claim amount; LHIC = Legitimare health insurance claim; T = Treatment; NB = New block; BCT = BlockChain **Begin**
 **Step 1.**
 HIN Commitment
    
$HIP\to ProvideDetails\left(HIN\right)\leftarrow HIS$
    
$HIS\to Purschase\left(HIN\right)\leftarrow HIP$
    
$HIP\to CommitTransaction\left(HIN\right)\leftarrow HIS$
    
$Generate\left(ST\right)\leftarrow CommitTransaction$
    
$Add\left(NB\right)\to BCT$

 **Step 2.**
 HIC Generation
    
$HIS\to Request\left(T\right)\leftarrow HSP$
    
$HSP\to Handle\left(T\right)$
    
$HSP\to Input\left(T\right)\to SHS$
    
HSP→Prepare(HIC)
    
$HSP\to Validate\left(HIC\right)\leftarrow HSP$
    
$HSP\to Forward\left(HIC\right)⇑\left(5G,E\right)\to HCl$
    
$Generate\left(ST\right)\to T$
    
$Add\left(NB\right)\to BCT$

 **Step 3.**
 HIC Processing
    
$HIP\to Recieve\left(HIC\right)$
    
$HIP\to Query\left(HIC,HCl\right)$
    
$HIP\to Apply\left(ST\right)\leftarrow HIC$
    
If(HIC∈LHIC)
      
$HIP\to Process\left(LHIC\right)$
      
$HIP\to Refund\left(CA\right)\to (HSP,HIS)$
    
else
      
HIP→Refuse(HIC)
    
HIP→SignTransaction()
    
$Add\left(NB\right)\to BCT$

***End***

### 3.6. HIC Processing Layer

The key stakeholders of this layer are HIPs, each of which can be a private or a governmental firm responsible for paying health services directly to the HINS or the HC. The HIN processing layer is connected with all the other layers of the proposed framework. It is associated with a storage layer to fetch HIC information from the healthcare cloud. It communicates with the prediction layer to obtain information about claim authenticity. It uses security layer features to ensure HIC authenticity through smart contracts. This layer uses 5G services of the communication layer for data fetching and transmission. This layer is also connected with the HC and HINS to inform them about the results of HICs.

## 4. Framework Evaluation

In this section, we will show the details of framework evaluation using various methods. We first evaluated the proposed framework using mathematical modeling. The feasibility of the framework was assessed with the help of a focus group by evaluating various parameters using concept mapping and Likert scale evaluation, and finally, a case study of wearable devices was used to further elaborate and evaluate the proposed framework. Below, we discuss these evaluation results in detail.

### 4.1. Mathematical Modeling

Below, we explain the evaluation and step-wise working of SHINFDP. Notations part in Abbreviations provides the list of mathematical notations used in the model.

Let the smart HIN process ℘ take patient data ${𝚰}_{\rho }$ as input, transmit it to the smart healthcare system $S_{h}$ from where it is stored on the healthcare cloud $H_{c}$, and the hash of the data is added in to the blockchain $B_{c}$. The machine learning algorithm $M_{l}$ is applied to this data to extract legitimate ${𝚰}_{C}$. The HIN provider $H_{p}$ tries to maximize the ℘ by only processing legitimate ${𝚰}_{C}$. Thus, our objective function will be as follows:

$Objective\;Function\;OF=\underset{0\leq x\leq n}{\max}℘(x)$where

(1) $$℘=\left\{{𝚰}_{\rho },S_{h},H_{c},G,M_{l},B_{c},{𝚰}_{C}\right\}⟹\sum_{c=1}^{n}\left\{\begin{array}{c} {𝚰}_{\rho }=true\\ L_{i}=true\\ F_{c}=false \end{array}\right.$$

Under the constraints of $\propto S_{h},M_{l},B_{c},G{,H}_{c}$

(2) $${𝚰}_{\rho }=\left(\begin{array}{ccc} V_{i} & \cdots & S_{h}\\ \vdots & \ddots & \vdots \\ T_{o} & \cdots & {𝚰}_{C} \end{array}\right)⋉\left[\begin{array}{ccc} H_{c} & \cdots & B_{c}\\ \vdots & M_{l} & \vdots \\ G & \cdots & L_{i} \end{array}\right]$$

According to Equation (2), patient data input ${𝚰}_{\rho }$ will be validated by first checking that the patient’s record exists in $S_{h}$ and that he/she has a valid insurance policy; after that, he will be provided the required treatment, and after obtaining treatment $T_{o}$, insurance claim ${𝚰}_{C}$ will be made. This ${𝚰}_{C}$ needs to be stored on the healthcare cloud $H_{c}$ using G, and the corresponding hash value will be added to the new block of the blockchain $B_{c}$ where the machine learning algorithm will be applied to extract legitimate HIN claim $L_{i}$.

(3) $$S_{h}(x)=\left\{\begin{array}{c} H_{c},x\;is\;true\\ G,x\;is\;true \end{array}\right.$$

According to Equation (2), in order to make ℘ a successful, smart healthcare system, $S_{h}$ need to be functional, and this is only possible when it is connected to $H_{c}$ using G.

(4) $$H_{c}=\left[\begin{array}{ccc} {𝚰}_{C} & \cdots & E_{c}\\ \vdots & \ddots & \vdots \\ T_{o} & \cdots & O_{X} \end{array}\right]⨂\left[\begin{array}{ccc} O_{X} & \cdots & B_{c}\\ \vdots & \ddots & \vdots \\ D_{l} & \cdots & H \end{array}\right]$$

According to Equation (3), for smart and secure ℘, healthcare cloud $H_{c}$ needs to work properly, which will be accomplished if and only if ${𝚰}_{C}$ and $T_{o}$ are encrypted and claim data are stored $O_{X}$. Further security will be implemented by adding the hash H of the stored data on the blockchain digital ledger $D_{l}$ and creating a new block in the blockchain $B_{c}$.

(5) $$G=f\left(x\right)=\left\{\begin{array}{l} S(x),x\;is\;maximum\\ L(x),x<x\;is\;minimum \end{array}\right.$$

For efficient ℘, the transmission of ${𝚰}_{\rho }$ at maximum speed $S\left(x\right)$ and minimum latency L(x) is required, which is only possible if G is implemented and working properly, as shown in Equation (5).

(6) Ml=OX⇒Hc⇔H⇔G𝚰ρ⋯Li⋮⋱⋮Dc⋯Fc

The stored claim data $O_{X}$ will be fetched from $H_{c}$ via G by ensuring its security using H. This input data will be decrypted and using suitable ML techniques it will be classified into legitimate $L_{i}$ and fraudulent claim $F_{c}$, as shown in Equation (6).

(7) Bc=→yieldsfx=Dl=Dl+𝕳,x=0Dl=0∩+𝕳,x≥0CmSc

According to Equation (7), the blockchain digital ledger $D_{l}$ will add the hash of the data by executing $C_{m}$ and will generate a $S_{c}$ that will be populated by all the entities involved to secure ${𝚰}_{\rho }$ and $O_{X}$.

(8) $${𝚰}_{C}=\left[\begin{array}{ccc} T_{o} & \cdots & H_{p}\\ \vdots & \ddots & \vdots \\ {𝚰}_{\rho } & \cdots & H_{c} \end{array}\right]\overset{yields}{\to }\underset{F_{c}}{\underbrace{L_{i}}}$$

The insurance claim will be validated by $H_{p}$ with the help of patient input data ${𝚰}_{\rho }$, the obtained treatment $T_{o}$, and through the data stored on $H_{c}$, that will yield the result of legitimate HIN claims $L_{i}$ by identifying and reporting $F_{c}$, as shown in Equation (8).

(9) $$\underset{0\leq x\leq n}{\max}℘\left(x\right)=\left\{\begin{array}{c} {𝚰}_{\rho }\underset{H_{c}}{\Rightarrow }\cap \left(\begin{array}{cc} D_{l} & H\\ C_{m} & S_{c} \end{array}\right)\\ S_{h}\overset{∆}{\to }H_{c}\{H_{p},H_{s},H_{r}\}\\ \underset{{𝚰}_{\rho }}{\max}G\overset{∆}{\to }H_{c}\left(\begin{array}{cc} D_{l} & H\\ C_{m} & S_{c} \end{array}\right) \end{array}\right.$$

**Corollary.**  *The Security of the* ℘ *can be compromised if* $F_{c}$ *is generated and not detected by* ℘*. Furthermore,* $H_{p},H_{s}\;or\;H_{r}$ *may be involved in HIN fraud. The* $F_{c}$ *is immediately detected, and any manipulation in* $I_{C}$ *is prohibited using the SHINFDP*.

**Proof.**  Assume that a $H_{p},H_{s}\;or\;H_{r}$ intends to manipulate ${𝚰}_{C}$. The value of H is immutable, so it does not allow this change and declares the altered block as $F_{c}$. This will prevent $H_{p},H_{s}\;or\;H_{r}$ from manipulating ${𝚰}_{C}$. The $S_{c}$ between $H_{s}$ and $H_{p}$ also keeps both parties on the same pace and does not allow anyone to violate the $S_{c}$. Below, we discuss the possible ways of disrupting the security and integrity of ℘ and how the proposed SHINFDP helps to save the ℘ from security breaches.

1. $H_{p}$ Breaches: Whenever $H_{p}$ tries to deliberately delay or manipulate the ${𝚰}_{C},$ the $S_{c}$ between $H_{p}$ and $H_{s}$ helps to solve this problem by providing a copy of the transparent agreement between both parties.
2. $H_{s}$ Breaches: Whenever $H_{s}$ tries to hide its identity or misuse his $V_{i}$ to obtain illegal benefits, the $O_{X}$, $C_{m}$ and $S_{c}$ hinders his way and thus prevents any kind of fraudulent ${𝚰}_{C}$ execution.
3. $H_{r}$ Breaches: If $H_{r}$ tries to manipulate ${𝚰}_{C}$, the $H_{c}$ data that is stored using
  $E_{c}$ and is connected to the $B_{c}$ using H becomes disconnected from existing
  $B_{c}$, which immediately highlights the fraudulent activities.

The above discussion shows that properly implementing SHINFDP makes the HIN process fair and transparent. Furthermore, the proposed SHINFDP provides flexibility to the stakeholders in selecting cloud services and ML algorithms according to their choice. The limited and expensive storage of the blockchain DTL can be overcome by storing HIN data on the cloud and just saving the hash of the data on DTL. □

### 4.2. Industrial Evaluation

A focus group technique was used to evaluate the ease of use, security, understanding, and structure of the proposed SHINFDP. Numerous factors, such as cost-effectiveness, observation of body language, and enhanced consumer engagement, contribute to the selection of the focus group technique for evaluation. Focus groups are comparatively inexpensive to conduct, and the moderator is able to observe the interviewee and modify the conversation accordingly. It is also more engaging than impersonal research methods such as questionnaires and surveys. However, sizable samples are difficult to target when using focus groups. To measure the ease of use, security, and structure of the SHINFDP, closed-end questions were designed and asked to the focus group respondents. In contrast, the concept mapping technique was used to evaluate the understanding of the SHINFDP. Seven professionals were included in the focus group, from which four belonged to a well-known HIN provider company in Saudi Arabia, while three of them were healthcare professionals dealing with insurance claim handling in a well-known public sector hospital. Below, we describe the details of each measure used to evaluate SHINFDP.

#### 4.2.1. Evaluating Ease of Use for SHINFDP

A desktop meeting was arranged with respondents of the focus group in which the author presented the workings of the SHINFDP to the respondents via PowerPoint presentation. At the end, the respondents were asked five closed-ended questions to evaluate the feasibility of the SHINFDP. Table 1 shows the responses of focus group members where SA refers to strongly agree, A→agree, D→disagree, SD→strongly disagree, and N→Neutral. The threshold value was set to 50%, which means that if more than 50% of the respondents agree on any statement, it will be considered acceptable. The total was calculated by adding the value of SA and A.

**Figure 6:**
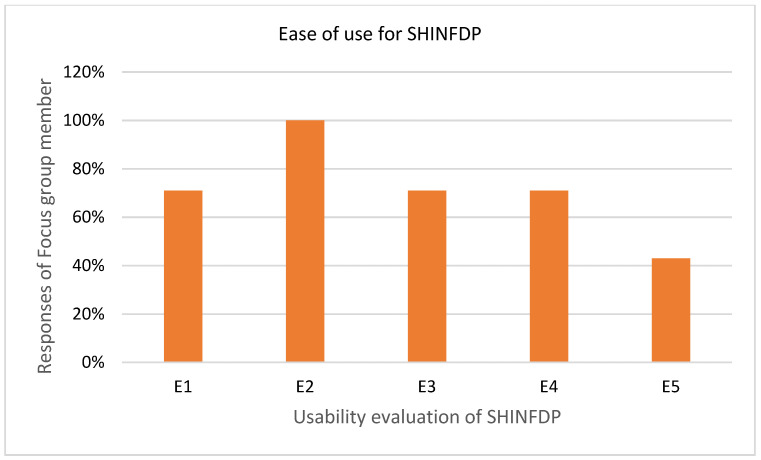
SHINFDP evaluation regarding “ease of use” criteria.

Figure 6 depicts the respondents of the focus group’s opinions regarding the usability features of SHINFDP. E1, E2, E3, and E4 were positive statements, in which a higher agreement percentage refers to more usability, while E5 was negatively assessing the usability of SHINFDP. Figure 6 shows that the agreement percentage of E1, E2, E3, E4 is more than the threshold value, while the agreement percentage of E5 is 43%. This shows that SHINFDP is easy to use and is adaptable by healthcare organizations.

#### 4.2.2. Evaluating the Security Features of SHINFDP

Using the same pattern mentioned in “ease of use” evaluation, respondents of the focus group were asked five close-ended questions for the security evaluation of SHINFDP. The questions and responses are given in Table 2, along with the total and average percentage of agreement.

**Figure 7:**
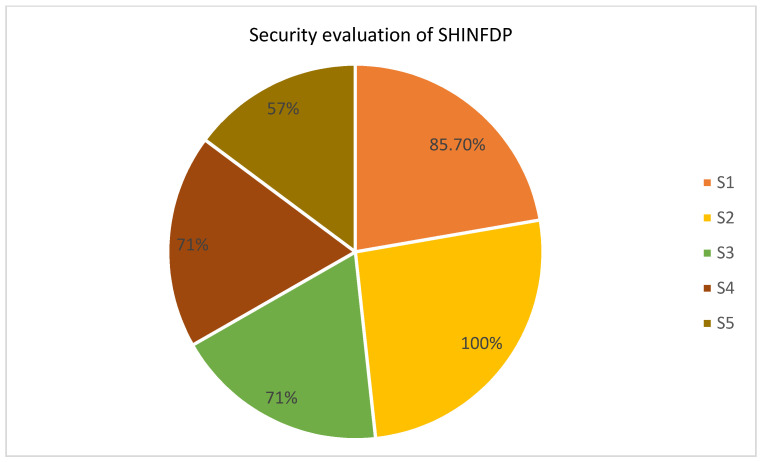
Security evaluation of SHINFDP.

The security evaluation results are depicted in Figure 7; the agreement percentage regarding the five mentioned elements was higher than the threshold value. This shows that SHINFDP helps to improve the security of the HIN process.

#### 4.2.3. Evaluating Understanding Feature of SHINFDP

**Figure 8:**
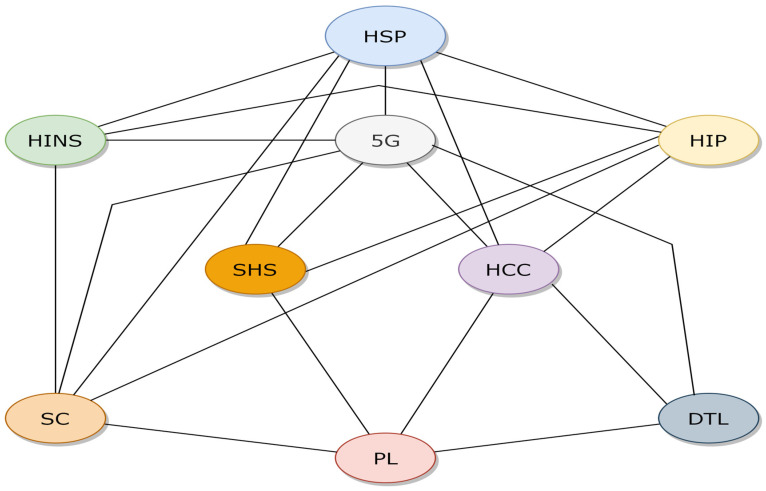
A partial concept map of the SHINFDP.

In order to evaluate the understanding level of respondents regarding SHINFDP, a concept mapping technique was used. Concept maps are diagrams that express information visually. Charts, graphic organizers, tables, flowcharts, Venn diagrams, timelines, and T-charts are a few examples of how they might be presented. Concept maps are particularly helpful for assessing how well a phenomenon is understood. Figure 8 shows the example concept map for the SHINFDP, which shows how various components of the SHINFDP are related.

In order to evaluate the focus group respondents’ understanding of SHINFDP, we extracted eight relationships between concepts and asked the respondents to evaluate whether this relationship should exist or not. Table 3 shows the selected relationships and respondents’ responses.

Table 3 shows that the respondents’ evaluation of SHINFDP is more than 70% for all relationships. This shows that SHINFDP is easy to understand for healthcare professionals.

#### 4.2.4. Evaluating the Structure of SHINFDP

SHINFDP is a layered framework consisting of six layers, and different cutting-edge technologies are proposed at various layers. Three questions were used to evaluate the structure of SHINFDP which are given in Table 4.

**Figure 9:**
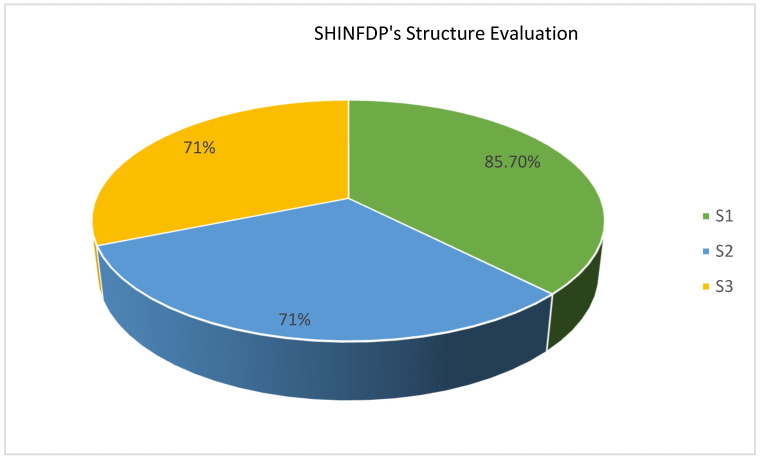
Respondents’ evaluation regarding the structure of SHINFDP.

Figure 9 depicts the responses of focus group respondents regarding the evaluation of SHINFDP’s structure. The agreement percentage for all three questions was more than 70 percent, showing that SHINFDP has a simple and easy-to-understand structure.

### 4.3. Evaluation Using a Case Study

Because of the COVID-19 epidemic, individuals have become more sensitive to positive living choices. They have started adopting healthier lifestyles using cutting-edge technologies such as wearable devices (WRD) and smartphone healthcare applications. Wearable technology can be any electronic device worn on the user’s body [57,58,59]. Smartphone healthcare applications provide medical-related services. Clinical research may take advantage of the data collected by healthcare applications and WRDs. Some HIP businesses utilize the data collected from WRDs for future predictions [60,61]. They employ WRDs as part of their marketing strategy to engage with HINSs. HIP enterprises may give prizes or discounts on premium pricing and free WRDs under specific situations as part of their marketing strategy. Conditions include the HINS using a WRD, providing authorization for WRD tracker data sharing, and demonstrating healthy habits such as walking a specific amount of steps each week, counting calories, and keeping a healthy heart rate using WRD [62]. In 2016, a US-based HIN startup established a platform to inspire and reward HINSs to partake in healthy activities using a WRD. According to data collected from WRDs, the company offers HINSs challenges and competitions, and in return, HINSs receive workout equipment, gift vouchers, and lower HIN insurance rates [54]. Another US-based partner company provides free MI-fit fitness trackers to all HINSs. This business is one of the first HIP companies to analyze insurance premium rates for HINSs using WRD data. They connected their HIN account to the HINS’s biometric data [54]. An HIN corporation located in France encourages HINSs to exchange health-related personal data from a WRD. By sharing their WRD data, HINS may earn fit points. This information is used in the firm’s insurance underwriting procedure [63]. An Australian-based HIP startup also collaborated to incorporate HINS data from different WRD trackers into their accounts. This information evaluates HIN risks and sets rates [64]. As previously stated, the HIN business utilizes WRD data to evaluate premium pricing and insurance risk assessment; however, it has not yet been used for HIN fraud detection.

**Figure 10:**
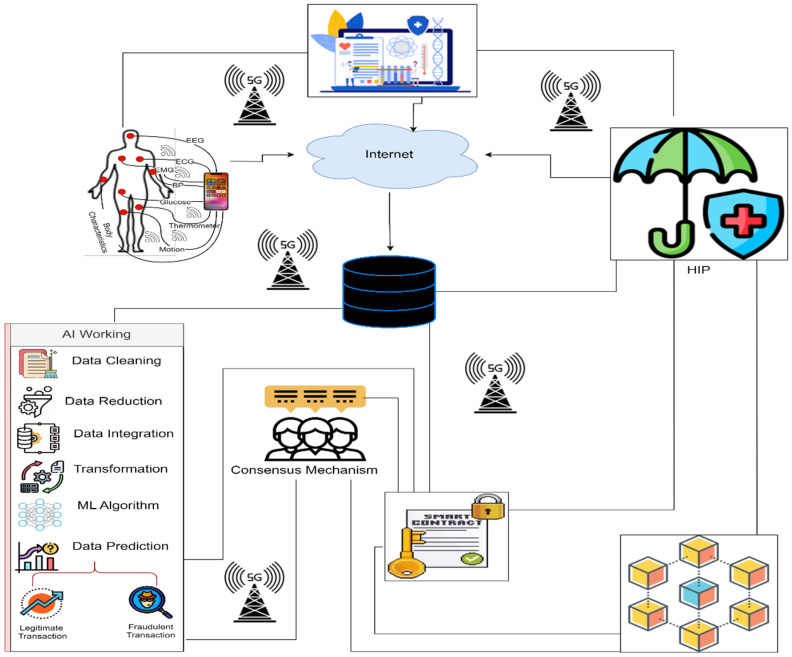
A case study of HIC fraud detection.

HIN fraudsters become smarter daily and develop new methods to commit fraud. As a result, every HIN company must discover a new and enhanced future strategy to prevent fraud utilizing a WRD’s data. As a result, we provide a case study to meet the criteria mentioned above for HIN companies. Figure 10 depicts the working of the case study that addresses HIN security and privacy concerns. The HINS is wearing several WRD/sensors in Figure 10. The HIN company may provide the WRD for marketing and fraud detection in HIC. The HINS WRD communicates with HINS smartphones through Wi-Fi or Bluetooth. The data produced by the WRD is encrypted and digitally signed before being saved in IPFS. The data from the HIP and HSP is encrypted, digitally signed, and transferred to the fraud detection system. Data preparation follows data production and involves data cleansing, integration, transformation, and reduction methods. The HINS distributes permission for sharing personal healthcare data using a preset smart contract before data preparation. The HIP business provides the public key to the HINS and HSP. If an HINS approves exchanging personal health data, the HIN corporation will reward the HINS. An AI algorithm is used to preprocess the data to determine if the HIC is fraudulent or authentic. If a claim is genuine, the BLC network consensus permits automatic HIC coverage payment to the HINS or HSP. However, if the HIC is false, the HINS will pay a penalty.

The use of WRDs in a HIC has various advantages. It enhances the HINS’s involvement with the HIP regularly and in real time. The WRD will be used to track the HINS’s whereabouts. HINS location data and real-time health status may be used to determine what occurred before, during, and after a hospitalization emergency. HIN corporations may use this data to identify pain levels, prevent medication-seeking behavior, and uncover inconsistent conduct with a HIC. Furthermore, BLC technology provides new avenues for improving inefficiencies in the HIN market. It may benefit HIPs by improving security, lowering costs, and increasing confidence. BLC in the HIN business also offers real-time data that may be utilized for risk evaluation, claims settlement, and other purposes.

Based on the case study and existing state-of-the-art review, it can be concluded that a combination of cutting-edge technologies, including BLC, 5G, cloud, AI, and wearable devices, can improve the HIC process and make it more transparent. When HIC fraud reduces, HIP firms will benefit from it, and HINSs will also benefit in the form of lower HIN premium amounts. Academia and practitioners should provide easy solutions for implementing these solutions in reality so that more people around the globe could benefit from healthcare facilities.

### 4.4. Discussion

A reduced mortality rate, better health outcomes, and increased productivity are all related to having health insurance. Health insurance also makes it easier to receive treatment. Even though it has many advantages, many people still do not have health insurance, which puts their physical, emotional, and financial health in danger. One of the challenges that the HIN procedure must contend with is fraud with the HIN. In this sort of fraud, a health insurance company is given information that is either false or deceptive to trick them into paying unauthorized benefits to the policyholder, another party, or the organization delivering services. It is possible for the insured person or the supplier of health services to commit the offence. The majority of fraud is conducted by HINSs and HSPs; however, HIPs are also guilty of conducting fraud on occasion, although their involvement in fraud is often limited to intentionally delaying the processing of claims or artificially understating their value. We presented a six-layered framework that utilizes the potentials of cutting-edge technology for safeguarding healthcare data and combating HIN fraud in order to make the HIN process smart and secure. An assessment of the suggested framework was carried out with the assistance of mathematical modelling, an evaluation of the industry that made use of the focus group approach, and a case study. The outcomes indicate that the suggested framework is straightforward, useful, secure, and adaptable to various circumstances.

The proposed approach is different from the existing solutions [5,15,24,53,54,55,56] in the following ways:

- Existing solutions mainly focus on a particular technology and try to determine its potential in improving the HIN process;
- Existing solutions discuss the benefits of using BLC in the HIN process, but they do not evaluate the solution due to the limitation of there being less use of BLC in healthcare;
- The existing studies used a single method of evaluation, while the proposed solution is evaluated using hybrid approach (mathematical modeling, focus group study, and industrial case study), which shows the generalizability of research findings;
- Existing studies mainly emphasize the adaptability of the HIN process and provide various techniques for it. Furthermore, there exist no solutions that specifically address the end-to-end HIN process cycle security.

### 4.5. Limitations of the Study

Below are some limitations of this research work.

1. We evaluated the proposed framework SHINFDP using a focus group technique in which only seven professionals were involved. However, we tried to choose a representative sample.
2. The use of mentioned technologies will add to the cost for HINP and HSP, but the enormous costs associated with insurance fraud will be avoided. The use of cutting-edge technology requires expenses in terms of hardware and training, but it is a one-time investment on the part of healthcare organizations and HINP, with long-term rewards. Other advantages of this automation will include convenience for the parties engaged in the HIN process, no need for manual visits, fast communication between health insurance subscribers and health insurance providers, audit and checking, lack of fraudulent activities, easy claim verification, and many more that will also indirectly save on costs.
3. ML and cloud computing are widely used in healthcare for prediction and storage. However, blockchain technology is somewhat new and is not commonly adopted in the HIN process. Therefore, we could not implement the proposed framework in a real setting.

## 5. Conclusions and Future Work

We presented a comprehensive summary of HIN fraud detection and related security vulnerabilities in HIC. First, we discussed the background of HIN, including the HIN revolution, varieties of HIN fraud, and the HIC process. Then, we discussed the many parties in the HIC process and the accompanying fraudulent acts that lead to HIN fraud. We discussed the role of BLC and AI in HIN. Given the significance of HIN fraud, we presented SHINFDP, an end-to-end solution for safe HIC processing to prevent HIN fraud. SHINFDP is made up of six layers: the user layer, the communication layer, the storage layer, the security layer, the prediction layer, and the HIP layer. Each layer contributes to the improvement of the HIC process. The proposed framework was refined with the assistance of a case study. The results indicate that the proposed method may assist in identifying and mitigating HIN fraud and make the HIC process more transparent. In the future, we want to verify the suggested framework by deploying it in a real-world healthcare context or through simulation. The proposed framework might be improved further by using more cutting-edge tools and technologies to optimize the HIC process.

## Figures and Tables

**Table 1:** Evaluation results for “ease of use” of SHINFDP.

	Ease of Learning	SA	A	D	SD	N	Total	Mean
E1	Comprehending the information flow and the function of cutting-edge technologies in SHINFDP is straightforward.	2	4	1	0	0	5	71%
E2	It is easy to handle the end-to-end HIN process using SHINFDP	4	3	0	0	0	7	100%
E3	It is easy to use SHINFDP to assess organizations’ readiness for SHINFDP	2	2	1	0	2	4	71%
E4	Each individual technology is easy to understand and implement	2	3	1	0	1	5	71%
E5	Some training needs to be provided for the use of SHINFDP	1	2	2	1	1	3	43%

**Table 2:** Security evaluation of SHINFDP.

	Security	SA	A	D	SD	N	Total	Mean
S1	Using SHINFDP makes the HIN process end-to-end secure.	3	3	1	0	0	6	85.7%
S2	Multilevel security is provided in SHINFDP by first handling it at the healthcare cloud level using encryption and then using BLC.	5	2	0	0	0	7	100%
S3	It is difficult for any stakeholder to manipulate the HIN claim.	2	3	0	0	2	4	71%
S4	ML algorithms further improve security by filtering HIN claims.	3	3	0	0	1	5	71%
S5	5G makes the HIN process more secure	2	2	1	1	2	3	57%

**Table 3:** Evaluating relationships between concepts.

S.No	Relationship between Concepts	Yes	No	Percentage (Yes)
1	HIP $\overset{Approaches}{\to }$ HSP	7	0	100%
2	HSP $\overset{Uses}{\to }$ SHS	7	0	100%
3	BLC $\overset{have}{\to }$ DTL	6	1	85.7%
4	DTL $\overset{Keep\;Hash}{\to }$ HCC	6	1	85.7%
5	5G $\overset{Facilitate\;storage}{\to }$ HCC	6	1	85.7%
6	SC $\overset{Store\;info}{\to }$ HIP	5	2	71%
7	BLC $\overset{Maintain}{\to }$ SC	5	2	71%
8	PL $\overset{Fetch\;data\;for\;prediction}{\to }$ HCC	6	1	85.7%

**Table 4:** Evaluating the structure of SHINFDP.

S.NO	The Structure of SHINFDP	SA	A	D	SD	N	Total	Mean
S1	All the layers of the SHINFDP are self-explanatory and require no further explanation to be used effectively	4	2	1	0	0	6	85.7%
S2	SHINFDP is practical and applicable in the HIN industry	2	3	1	0	1	5	71%
S3	The distribution of technologies at various layers is correct	3	2	1	0	1	5	71%

## Data Availability

Not applicable.

## Abbreviations

**Acronyms**	**Used for**
AI	Artificial intelligence
BLC	Blockchain
DL	Deep learning
DTL	Digital ledger
HC	Healthcare center
HIC	Healthcare insurance claim
HIN	Healthcare insurance
HIP	Healthcare insurance provider
HCC	Healthcare cloud
SHINFDP	Smart healthcare insurance framework for fraud detection and prevention
CET	Cutting-edge technologies
IPFS	Interplanetary File System
ML	Machine language
WRD	Wearable devices
SHS	Smart healthcare system
SL	Supervised learning
SSL	Semi-supervised learning
USL	Unsupervised learning
HINS	Healthcare insurance subscriber
PL	Prediction layer
**Notations**	**Used for**
$I_{\rho }$	Patient data input
$T_{o}$	Obtain treatment
H	Hash/unique ID
$I_{C}$	Insurance claim
℘	HIN process
$S_{h}$	Smart healthcare system
$V_{i}$	Valid insurance
$H_{s}$	HIN subscriber
G	5G
$F_{c}$	Fraudulent claim
$L_{i}$	Legitimate $I_{C}$
$H_{c}$	Healthcare cloud
$M_{l}$	ML algorithm
$B_{c}$	Blockchain
$D_{l}$	Digital ledger
$C_{m}$	Consensus mechanism
$S_{c}$	Smart contract
$O_{X}$	Store claim data
$H_{r}$	Healthcare professional
$E_{c}$	Encryption
$D_{c}$	Decryption
$H_{p}$	HIN provider
